# The Immunological Synapse: An Emerging Target for Immune Evasion by Bacterial Pathogens

**DOI:** 10.3389/fimmu.2022.943344

**Published:** 2022-07-13

**Authors:** Nagaja Capitani, Cosima T. Baldari

**Affiliations:** Department of Life Sciences, University of Siena, Siena, Italy

**Keywords:** pathogens, immunological synapse, Antigen Presenting Cell (APC), major histocompatibility complex class II (MHCII), T cell receptor (TCR), actin cytoskeleton

## Abstract

Similar to other pathogens, bacteria have developed during their evolution a variety of mechanisms to overcome both innate and acquired immunity, accounting for their ability to cause disease or chronic infections. The mechanisms exploited for this critical function act by targeting conserved structures or pathways that regulate the host immune response. A strategic potential target is the immunological synapse (IS), a highly specialized structure that forms at the interface between antigen presenting cells (APC) and T lymphocytes and is required for the establishment of an effective T cell response to the infectious agent and for the development of long-lasting T cell memory. While a variety of bacterial pathogens are known to impair or subvert cellular processes essential for antigen processing and presentation, on which IS assembly depends, it is only recently that the possibility that IS may be a direct target of bacterial virulence factors has been considered. Emerging evidence strongly supports this notion, highlighting IS targeting as a powerful, novel means of immune evasion by bacterial pathogens. In this review we will present a brief overview of the mechanisms used by bacteria to affect IS assembly by targeting APCs. We will then summarize what has emerged from the current handful of studies that have addressed the direct impact of bacterial virulence factors on IS assembly in T cells and, based on the strategic cellular processes targeted by these factors in other cell types, highlight potential IS-related vulnerabilities that could be exploited by these pathogens to evade T cell mediated immunity.

## 1 Introduction

Successful microbial pathogens, such as bacteria, have evolved complex and efficient strategies to evade the host immune response. To establish chronic infection bacteria have to overcome the two powerful arms of the host immune defenses, innate and adaptive immunity. Innate immunity is evolutionarily conserved among higher eukaryotes and represents the first line of defense against infections, with the key role to recognize pathogen components and start the process of microbial clearance. Additionally, innate immune cells are central for the development of adaptive immunity. Hence, not surprisingly, pathogens have evolved a variety of mechanisms to elude this first line of the host immune defenses, from building a protective capsule (e.g. *Streptococcus pneumoniae*, *Haemophilus influenzae*, *Escherichia coli*, *Neisseria meningitidis*) (1), to interfering with recognition of Pathogen-Associated Molecular Patterns (PAMPs) by host Pattern Recognition Receptors (PRRs) such as Toll-like receptors (TLRs) and C-type lectin receptors (e.g. *Helicobacter pylori*) (2), to inhibiting phagocytic activity (e.g. *H. pylori*, *Yersinia pestis*) (3). Remarkably, evasion of innate immunity is often accompanied by the exploitation of innate immune cells such as macrophages, which have been incapacitated to kill internalized bacteria by specific virulence factors, as a protected niche for replication.

Another strategy deployed by several bacterial pathogens to escape the host immune system is to prevent the development of the exquisitely specific and highly effective adaptive response. Adaptive immunity involves a tightly regulated interplay among B lymphocytes, T lymphocytes and antigen presenting cells (APCs) to activate pathogen-specific and lifelong immunological effector pathways. The development of T cell mediated immunity relies on the assembly of a highly specialized signaling and secretory platform formed by T cells at the interface with cognate APCs, known as the immunological synapse (IS). In this minireview we will briefly review the strategies evolved by bacterial pathogens to suppress T cell activation and discuss emerging evidence that highlights the IS as a key target for pathogens to evade the T cell-mediated host immune response.

## 2 The Immunological Synapse

T cell activation is initiated in response to the interaction of the T cell antigen receptor (TCR) with antigenic peptides bound to major histocompatibility complex (MHC) molecules (pMHC) expressed on the surface of APCs, which participate in the cellular immune response by processing and presenting antigens for recognition by T lymphocytes. Antigen presentation is a complex multistep process, involving the processing of endogenous or exogenous pathogen-associated antigens, peptide loading on MHC, and localization at the cell surface of pMHC complexes which can interact with T cells expressing a cognate TCR. Bacterial antigen presentation is mainly mediated by MHC class II (MHCII) molecules found on the surface of professional APCs that present antigen-derived peptides to be recognized by CD4^+^ T cells.

Following TCR interaction with cognate pMHC, a specialized supramolecular structure, defined as immunological synapse (IS), forms at the T cell interface with the APC. IS formation requires not only TCR:pMHC interaction but also the accumulation of coreceptors, adhesion molecules, and signaling and cytoskeletal components at the T cell-APC contact area (4). In its mature configuration the IS features a peculiar “bull’s eye” architecture characterized by concentric domains, known as supramolecular activation clusters (SMAC), that differ in molecular composition and function (5). The central SMAC (cSMAC), mainly enriched in TCRs and TCR-associated proteins, is surrounded by the peripheral (pSMAC), enriched in integrins, such as lymphocyte function-associated antigen (LFA-1), and cytoskeleton-associated proteins. The pSMAC is in turn surrounded by the distal SMAC (dSMAC), which is enriched in F-actin as well as in molecules that are excluded from the IS centre due either to steric hindrance (e.g. CD43) or to their ability to negatively regulate signaling (e.g. CD45) (4). The dSMAC is also the IS domain where signaling starts with the assembly of TCR-CD28 microclusters that move centripetally towards the IS to eventually segregate to the cSMAC (6), where exhausted TCR are internalized to make room to new TCRs microclusters that continuously form at the periphery.

TCRs are associated not only with the plasma membrane, but also with recycling endosomes (7). Delivery to the synaptic membrane of this intracellular TCR pool is essential to replenish the plasma membrane pool as TCRs are internalized at the cSMAC, allowing for the steady inward flow of actively signaling TCR microclusters to sustain signaling for the extended timeframe required for T cell activation (7). This process is dependent on the polarization of the centrosome together with the secretory apparatus to the region beneath the T cell-APC contact (8), which sets the stage for polarized exocytosis. Polarized recycling from an intracellular vesicular pool is a strategy co-opted by a number of molecules that participate in IS architecture and function. These include surface receptors, such as the co-inhibitory receptor cytotoxic T lymphocyte antigen-4 (CTLA-4) (9), and intracellular signaling molecules, such as the lymphocyte-specific protein tyrosine kinase (Lck), the adaptor molecule LAT (10–12), and the small GTPase Rac1 (13).

**Figure 1:**
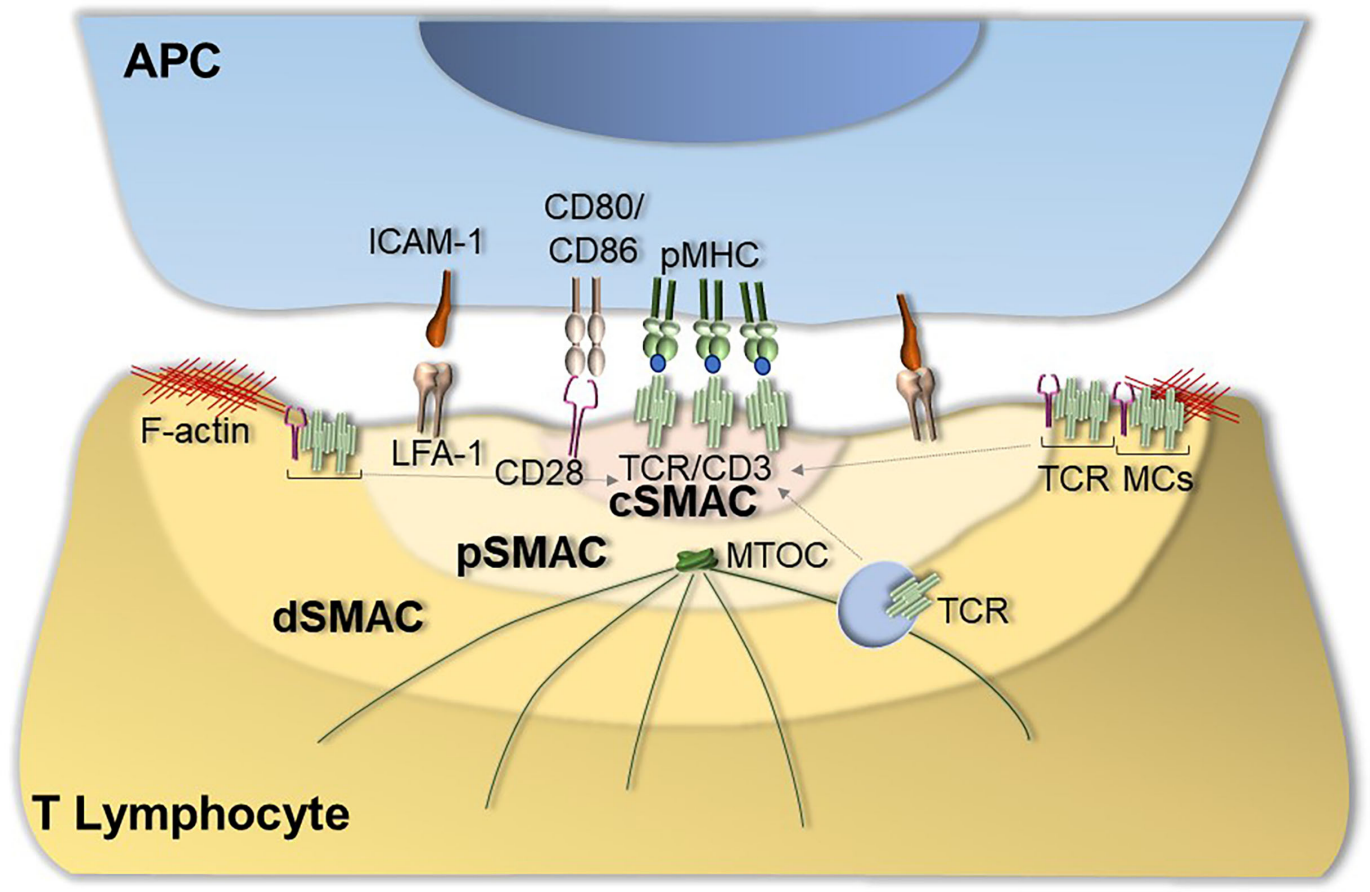
Immunological synapse assembly. The canonical IS shows a well-organized bull’s eye architecture that features the central supramolecular activation cluster (cSMAC) characterized by the presence of TCRs and TCR-associated proteins such as the co-stimulatory receptor CD28, the peripheral SMAC (pSMAC) enriched in the integrin LFA-1 and the distal SMAC (dSMAC) enriched in TCR-CD28 microclusters (TCR MCs) that move centripetally towards the cSMAC driven by F-actin. IS assembly is also coordinated by cytoskeletal dynamics that allow for centrosome translocation toward the IS as well as for the directional vesicular trafficking of receptors and signaling mediators to sustain signaling at the IS.

IS assembly is coordinated by the cytoskeleton (14–16), which plays a key role at different step of IS assembly, from integrin activation (16), to TCR microcluster movement from the periphery to the center of the IS (6), to centrosome translocation toward the IS (14), to the directional vesicular trafficking that ensures the continuous availability of receptors and signaling molecules at the IS (17–19) (**Figure 1**).

TCR interaction with pMHC at the IS triggers an intracellular tyrosine phosphorylation cascade, resulting in the activation of multiple signaling pathways. Briefly, the activated TCR recruits the initiating kinases Lck and ζ-associated kinase of 70 kDa (ZAP-70) which phosphorylates LAT, a multifunctional transmembrane adaptor that orchestrates the activation of phospholipase Cγ (PLCγ). By producing key second messengers, PLCγ promotes the activation of the PKC, Ras and Ca^2+^ pathways which couple TCR triggering to gene expression through the activation of transcription factors such as nuclear factor of activated T cells (NF-AT), nuclear factor-κB (NF-κB) and activating protein 1 (AP-1) (20).

As IS assembly is a key event for the development of T cell-mediated immunity, it is not surprising that many pathogens have developed virulence mechanisms to target IS formation, either indirectly by impairing the ability of APCs to present antigen to the T cell, or, as supported by emerging evidence, by directly inhibiting IS assembly within the T cell.

## 3 How Bacterial Infection Affects IS Assembly

### 3.1 Indirect Modulation of IS Assembly by Bacterial Pathogens Through APC Targeting

To initiate adaptive immunity to pathogens, T cells must interact with cognate APCs that have previously taken up antigen at the site of infection and have migrated to the draining lymph node. This role is subserved by dendritic cells (DCs) which are specialized for antigen presentation to naïve T cells, but in the context of bacterial infections it can also be taken over by macrophages. Several steps are required before an APC can acquire the appropriate functional status and be in the appropriate location to form an IS with a cognate T cell. These steps are orchestrated by innate immune receptors, which on recognition of bacterial PAMPs trigger the maturation of DCs, the phagocytic uptake and destruction of the pathogen, and the migration of the phagocyte to the closest lymph node station. As largely documented for viruses (1), also bacterial pathogens have evolved a variety of strategies to interfere with each of these steps, including camouflaging as host components (e.g. GAG proteins of *Streptococcus*), modifying PAMPs to decrease their potency in innate immune receptor activation (e.g. modified LPS core component lipid A of *Salmonella*) (21), inhibiting PRR signaling (e.g. *Salmonella* TIR domain-like TIpA to disrupt TLR4 signaling (22); *Yersinia* acetyltransferase YopJ to inhibit NF-kB signaling (23); *Mycobacterium tuberculosis* (*M. tubercolosis*) ubiquitin ligase PnkG to degrade components of the NF-kB-activating signalosome (24), or exploiting mimicry to activate inhibitory circuits (e.g. sialylated capsular polysaccharides of group B Streptococcus) (25, 26). For details on these upstream steps we refer the reader to excellent reviews (1, 27). Here we will focus on the process that is directly implicated in IS assembly -antigen presentation-, limiting the discussion to MHCII.

Antigen presentation to T cells by APCs plays an essential role in the initiation of adaptive immunity. As such, disruption of the process of antigen presentation is a mechanism co-opted by a number of bacterial pathogens to prevent the generation of specific effector T cells. Bacteria can modulate the MHCII pathway acting at different levels: by inhibiting MHCII gene transcription, by interfering with MHCII loading and trafficking, or by impairing antigen processing. The resulting defects in IS assembly translate into defects in T cell activation and differentiation to pathogen-specific helper T cell effectors. The intracellular pathogen *M. tubercolosis* is a remarkable example of how an individual pathogen can target the process of antigen presentation at every single level and we will use it as paradigm in the following sections.

#### 3.1.1 Inhibition of MHCII Expression

*M. tubercolosis* has the ability to potently downregulate MHCII expression, which occurs as part of the APC activation program triggered by PRR engagement. A well characterized *M. tubercolosis* factor implicated in this function is the 19-kDa lipoprotein (LpqH) which acts a potent TLR2 agonist. The resulting excessive or prolonged TLR2 activation leads to the expression of isoforms of the transcriptional transactivator C/EBP that inhibit the IFNγ-dependent induction of class II transactivator (CIITA), on which MHCII gene expression crucially depends (28, 29). Preventing MHCII upregulation to disrupt antigen presentation is shared by other *M. tubercolosis* virulence factors such as the cell envelope-associated serine protease Hip1 (30), and co-opted by a number of pathogenic bacteria (e.g. 31). One such example is *H. pylori*, which uses ADP183 heptose, an intermediate metabolite in LPS biosynthesis, to promote miR146b expression in macrophages, leading to downmodulation of CIITA expression (32).

**Figure 2:**
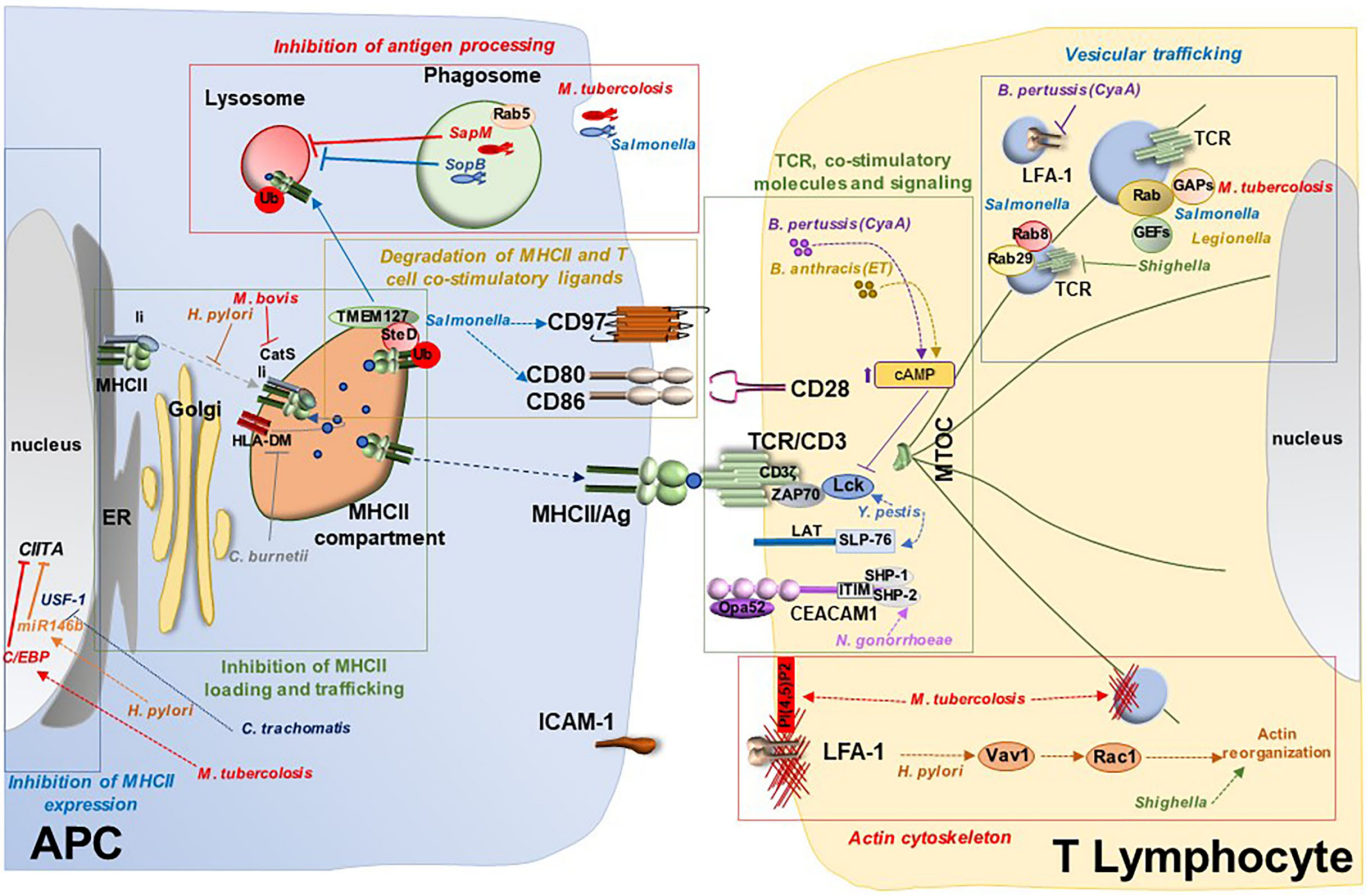
Bacterial targeting of the immunological synapse. Model for suppression of IS assembly by bacterial pathogens. Bacterial pathogens exploit a variety of virulence factors to interfere with IS assembly at different steps, both at the APC side and at the T cell side. Bacteria target APCs and hence indirectly IS assembly by interfering with different mechanisms: i) MHCII inhibition through modulation of transcription factors responsible for its expression (e.g. CIITA regulation by *M. tuberculosis, Helicobacter pylori* and *Chlamydia trachomatis*); ii) inhibition of antigen processing through suppression of phagolysosomal fusion (e.g. *M. tuberculosis* and *Salmonella)*; iii) defective antigen processing and loading onto MHCII in the MHCII compartment (e.g. inhibition of the Ii-dependent pathway by *Helicobacter pylori* or targeting CatS and HLA-DM by *M. tuberculosis* and *Coxiella burnetii*, respectively); iv) degradation of MHCII and T cell co-stimulatory ligands such as CD80/CD86 and CD97 (e.g. *Salmonella*). Bacteria interfere directly with IS assembly at the T lymphocyte side by i) targeting expression and function of the TCR and co-stimulatory molecules (e.g. CD3ζ degradation by *M. tuberculosis*, CEACAM1 disabling by *Neisseria gonorrhoeae* or impairment of TCR signaling by *Yersinia pestis, Bordetella pertussis* and *Bacillus anthracis*); ii) subverting the actin cytoskeleton (e.g. *Shigella flexneri*, *Yersinia pestis* and *Salmonella enterica* serovar *Typhimurium)*; and iii) interfering with vesicular trafficking by modulating Rab GTPases (e.g. *Salmonella enterica*, *Legionella pneumophila*, *Shigella*, *M. tuberculosis*) or by targeting receptor trafficking (e.g the TCR by *Shigella* or LFA-1 by *Bordetella pertussis*).

Whether the protease activity of Hip1 influences directly CIITA expression is not known. However, this mechanism has been documented for *Chlamydia trachomatis*, which secretes proteases that promote the degradation of the transcription factor USF-1 that regulates IFN-γ induction of CIITA expression (33). A different mechanism to lower MHCII expression is exploited by *Salmonella*, which induces surface MHCII internalization by promoting the expression of the E3 ubiquitin ligase MARCH1 and K63-linked MHCII ubiquitination. Internalized ubiquitylated MHCII molecules are subsequently degraded following routing to the endolysosomal system (34) (**Figure 2**, **Table 1**).

**Table 1:** Bacterial virulence factors that target directly or indirectly IS as.

Pathogens	IS targeting site	Vitulence factors	IS inhibition mechanisms	Ref.
***Mycobacterium tuberculosis***	**APC** **T cell**	LpqH, Hip1 SapM manLAM miR-106b-5p manLAM mycolactone SerB2 SapM NdK	MHCII expression (C/EBP, CIITA) Antigen processing (inhibition of phagolysosomal fusion) Antigen processing (inhibition of phagosome acidification) MHCII loading and trafficking (inhibition of CatS activity and expression) TCR and co-stimulatory molecules (CD3ζ degradation) Signaling at the IS (degradation of Lck, ZAP-70, LAT) Signaling at the IS (inhibition of TCR signaling) Actin cytoskeleton (modulation of F-actin filament assembly) Actin cytoskeleton (modulation of phosphoinositide signaling) Vesicular trafficking (recruitment of Rab proteins) Vesicular trafficking (Rab GAP)	28–30 35 35–38 39 40 41 42 35 43
***Mycobacterium bovis***	**APC**	IL-10	MHCII loading and trafficking (inhibition of CatS activity and expression)	44
***Chlamydia trachomatis***	**APC**	proteases	MHCII expression (INF-γ, USF1, CIITA)	33
***Salmonella enterica***	**APC** **T cell**	pH regulation SopB SteD SopB, SopE, SptP SopB SopD2 GtgE	MHCII surface expression (E3 ubiquitin ligase, MARCH1, K63-linked MHCII ubiquitination) Antigen processing (inhibition of phagosome maturation) Degradation of MHCII (ubiquitylation) Inhibition of T cell co-stimulatory ligands (CD86/B7-2, CD97) Actin cytoskeleton (Rho GEF mimics, GAP mimics) Vesicular trafficking (SopB recruitment of Rab proteins) Vesicular trafficking (Rab GAP) Vesicular trafficking (inhibition of polarized TCR recycling)	34 45 46 47, 48 49–51 52 53 54
***Helicobacter pylori***	**APC** **T cell**	ADP-heptose VacA VacA	MHCII expression (miR146b, CIITA) MHCII loading and trafficking (inhibition of the Ii-dependent pathway) TCR and co-stimulatory molecules (suppression of TCR signaling, Ca^2+^-calcineurin pathway, dysfunctional MAP kinase network) Actin cytoskeleton perturbation	32 55, 56 57, 58 57, 59
***Coxiella burnetii***	**APC**		MHCII loading and trafficking (alteration of MHCII/HLA-DM interaction)	60
***Pneumococcus pneumonia***	**T cell**		TCR and co-stimulatory molecules (downregulation of CD28, ICOS, CD40L)	61
***Staphylococcus aureus***	**T cell**	SEA, SEB, SEE toxins	TCR and co-stimulatory molecules (massive T cell activation)	62
***Neisseria gonorrhoeae***	**T cell**	Opa52	TCR and co-stimulatory molecules (CEACAM1 suppression by phosphatases)	63
***Yersinia pestis***	**T cell**	YopH YopE, YopT	TCR and co-stimulatory molecules (dephosphorylation of TCR signalosome) Actin cytoskeleton (GAP mimics, modulation of GTP- GDP-bound forms of Rho GTPases)	64–67 68, 69
***Bordetella pertussis***	**T cell**	CyaA	TCR and co-stimulatory molecules (suppression of TCR signaling, cAMP)	70–72
***Bacillus anthracis***	**T cell**	edema toxin	TCR and co-stimulatory molecules (suppression of TCR signaling, cAMP)	73
***Clostridium botulinum***	**T cell**	C3 toxin	Actin cytoskeleton (modulation of GTP- GDP-bound forms of Rho GTPases)	74
***Shigella flexneri***	**T cell**	IcsA IpgD unidentified T3SS effector VirA, IpaJ	Actin cytoskeleton (modulation of F-actin filament assembly) Actin cytoskeleton (inhibition of cell chemotaxis) Actin cytoskeleton (inhibition of IS assembly) Vesicular trafficking (Rab GAP, inhibition of the polarized recycling of TCR-containing endosomes)	75 76 77 77, 78
***Listeria***	**T cell**	ActA	Actin cytoskeleton (modulation of F-actin filament assembly)	79
***Legionella pneumophila***	**T cell**	LepB Lgp0393, DrrA/SidM	Vesicular trafficking (Rab GAP) Vesicular trafficking (Rab GEF)	80 81, 82

#### 3.1.2 Inhibition of Antigen Processing

Pathogenic bacteria can modulate the MHCII pathway by inhibiting the fusion of the phagosome containing internalized bacteria with the lysosome, which not only allows escape from killing but leads to impaired antigen processing. Again, using *M. tubercolosis* as paradigm, inhibition of phagolysosomal fusion has been shown to involve retention of the early endosome marker Rab5 at the phagosomal membrane, with concomitant exclusion of the lysosome marker Rab7 (83), which results in a delay in phagosome maturation and defective antigen processing. *M* ***. tubercolosis*** targets this process by using its lipid phosphatase SapM to hydrolyze the phospholipid PI3P, which is essential for phagosome-late endosome fusion (35). Similarly, *Salmonella* blocks phagosome maturation by modulating the phosphoinositide composition of the *Salmonella*-containing vacuole through its lipid phosphatase SopB (45). Hence inhibition of phagosome maturation is co-opted by many pathogenic bacteria to prevent antigen processing while escaping killing.

An alternative strategy used by *M.* tubercolosis for disrupting antigen processing is inhibition of phagosome function. One of the underlying mechanisms involves a M. tubercolosis-derived lipid, the mannose-capped form of lipoarabinomannan (manLAM). manLAM blocks phagosome acidification by reducing the local recruitment of the tethering molecule EEA1, which is essential for delivery of lysosomal hydrolases to the phagosome (36). The failure of EEA1 to associate with the phagosome in M. tubercolosis-infected cells is caused by defective production of PI3P at the phagosome membrane due to defective Ca^2+^-dependent activation of the PI3K component VPS34 (35, 37, 84). Additionally, the transport of vacuolar ATPase (v-ATPase), which is essential for phagosome acidification and activation of lysosomal hydrolases, is impaired in *M. tubercolosis*-infected cells due to dephosphorylation of the VPS33B component of the v-ATPase sorting complex by the *M. tubercolosis* phosphatase PtpA (38) (**Figure 2**, **Table 1**).

#### 3.1.3 Inhibition of MHCII Loading and Trafficking

An alternative strategy exploited by a variety of pathogens to inhibit antigen presentation is to interfere with MHCII loading and trafficking. Macrophage infection with *M. bovis* leads to the inhibition of both activity and expression of the cystein protease cathepsin S (Cat S) (44), which mediates the late cleavage steps of the invariant chain (Ii) cleavage (85) required for the generation of MHCII molecules that can be efficiently loaded with peptide antigens and delivered to the cell surface. The defect in CatS expression has been ascribed to the *M. bovis*-dependent induction of the suppressive cytokine IL-10 which blocks Cat S gene expression (44) as well as of *M. tubercolosis* microRNA miR-106b-5p which downregulates its transcript (39).

Other bacterial pathogens target the key steps of MHC loading and trafficking to suppress the initiation of T cell response. This is the case of *H. pylori* which, through its major virulence factor Vacuolating cytotoxin A (VacA), interferes with the proteolytic generation of T cell epitopes that are loaded onto newly synthesized MHCII molecules, specifically inhibiting the Ii-dependent pathway (55). In addition, MHCII molecules are retained in the *H. pylori*-containing vacuoles in *H. pylori*-infected DCs, such that their trafficking to the cell surface is prevented (56). *Coxiella burnetii* impairs antigen presentation at a different step -loading of peptide antigen- by altering the interaction of MHCII with HLA-DM, a key step required for displacing from MHCII the Ii CLIP peptide to allow for loading of pathogen-derived peptides and transport to the plasma membrane of functional pMHC complexes. In *C. burnetii*-infected cells MHCII molecules fail to dissociate form HLA-DM and accumulate in enlarged intracellular compartments (60) (**Figure 2**, **Table 1**).

#### 3.1.4 Degradation of MHCII and T Cell Co-Stimulatory Ligands

An alternative mechanism for reducing the levels of pMHC complexes at the APC surface has been reported for *Salmonella*. This function is mediated by the type 3 secretion system effector SteD. This transmembrane protein forms a complex with mature endosome-associated MHCII molecules and the transmembrane host tumor suppressor TMEM127, a Nedd4 family E3 ubiquitin ligase adaptor. TMEM127 recruits the E3 ligase Wwp2 to the complex, inducing ubiquitylation of MHCII for subsequent lysosomal degradation (46). Interestingly, SteD exploits this degradation-promoting activity to reduce the expression of important T cell activating ligands expressed on APCs, including CD86/B7-2 which activated the key co-stimulatory receptor CD28 (47), and the plasma membrane protein CD97 that is required to stabilize the IS formed with T cells (48) (**Figure 2**, **Table 1**).

### 3.2 Direct Targeting of the T Cell IS by Bacterial Pathogens

Since the seminal discovery that lymphotropic viruses such as HIV-1 and HTLV-1 not only exploit the IS to evade the T cell response but apply the same building principles to form the virological synapse, a platform for cell-to-cell transmission, the IS has attracted major interest as a target for immune evasion by viral pathogens (86, 87). Whether and how bacterial pathogens can subvert IS assembly to avoid T cell immunity not indirectly by modulating DC activation and function, but directly, are questions that are only beginning to be formulated. DCs are present at the sites of infection where they can readily recognize pathogens through their wide array of PRRs, orchestrating a sophisticated response that not only optimizes their antigen presentation capacity but also provides all the signals that T cells require to differentiate to the most appropriate type of effector. At variance, T cells continuously cycle between blood and lymph and are activated in secondary lymphoid organs, where DCs migrate following pathogen recognition. However, a number of bacterial virulence factors are released as soluble factors that can be transported through the lymph to the closest lymph node station, where they can interact with naïve T cells and even enter them while not establishing a productive infection, as exemplified by the T cell delivery of Shigella T3SS effectors (88). Importantly, following their differentiation, effector T cells, whether CTLs or Th cells, are recruited to the site of infection to coordinate a combined attack with innate immune cells against the invading pathogen. There, effector T cells become a very relevant target for immune evasion.

Examples of IS targeting by bacterial pathogens are as yet very few. However, the substantial body of information acquired on how bacteria subvert pivotal cellular processes in host cells, such as cytoskeletal dynamics and vesicular trafficking, which are essential for IS assembly, suggests that we are looking at the tip of the iceberg. In this section we will present arguments to support this notion, discussing specific instances that provide experimental evidence that the IS is exploited not only by viruses, but also by bacteria, to evade T cell-mediated immunity.

#### 3.2.1 Targeting the TCR and Co-Stimulatory Molecules

A strategy that mirrors at the T cell side what we described above on the APC side is downregulation of TCR expression, as exemplified in Pneumococcus-related sepsis. Of note, T cells from these patients also coordinately downregulated the expression of the major co-stimulatory receptors CD28, essential for T cell activation, and ICOS and CD40L, required for T cell-dependent B cell maturation (61). A different mechanism to modulate CD3 expression is exploited by *M. tubercolosis*, involving degradation of its key component CD3ζ. This is achieved through upregulation of the E3 ubiquitin ligase Grail by manLAM (40). Although not tested directly, downregulation of surface TCR is expected to impact on IS assembly and local signaling, as witnessed by primary immunodeficiency disorders with CD3 deficiency (89).

*Staphylococcus aureus* uses the amply characterized mechanism of forced, antigen-independent TCR binding to MHCII mediated by its toxins SEA, SEB and SEE to promote massive T cell activation and inflammatory cytokine production associated with defective anti-bacterial T cell response. These toxins are able to elicit IS assembly with high efficiency and are in fact used as surrogate antigens to study IS assembly in polyclonal T cells. Interestingly, a different mechanism involving the *Staphylococcus* superantigens SEA, SEB and TSST-1, has been recently reported, based on cross-linking the co-stimulatory receptor CD28 with its ligand B7.2 on APCs (62). Since CD28 co-localizes with the TCR at the cSMAC, this double locking action is expected to lead to the generation of hyperstable and hyperactive immune synapses.

Another example of co-inhibitory receptor targeting for T cell suppression is CEACAM1 disabling by *Neisseria gonorrhoeae*. CEACAM1 is expressed as two isoforms differing in the length of its intracellular domain, with the long isoform endowed of two immunoreceptor tyrosine-based inhibitory motifs (ITIM). The gonococcal protein Opa52 interacts with CEACAM1 on CD4^+^ T cells, leading to phosphorylation of its ITIM motifs and recruitment of the tyrosine phosphatases SHP-1 and SHP-2, which dampen TCR signaling (63). A similar strategy to suppress CD4^+^ T cell activation is exploited by *Fusobacterium nucleatum*, *Neisseria meningitidis*, *Moraxella catarrhalis*, and *Haemophilus influenzae*, which also trigger CEACAM1 activation through specific adhesins (90–92). At variance, CEACAM1 has been recently reported to also act as a co-stimulatory receptor essential for the activation and proliferation of CD8^+^ T cells, preventing their exhaustion and promoting their antiviral activity (93). Interestingly, CEACAM1 engagement leads to the recruitment of Lck to the TCR and stabilizes this key initiating kinase at the IS (93). This finding underscores the IS as a potential important target of bacterial pathogens that produce CEACAM1 ligands (**Figure 2**, **Table 1**).

#### 3.2.2 Targeting Signaling at the IS

Major bacterial pathogens have the potential to target signaling downstream of the TCR, thereby affecting IS assembly and stability. *M. tubercolosis* exploits the manLAM-dependent upregulation of Grail mentioned above for CD3ζ downregulation to coordinately promote the degradation of essential mediators of the TCR signaling cascade, including the initiating tyrosine kinases Lck and ZAP-70, and the adaptor LAT required for signal amplification and diversification (40). Again, deficiency of these signaling mediators in experimental systems or primary immunodeficiencies supports the potential negative impact of *M. tubercolosis* in IS assembly. Another *M. tubercolosis*-derived molecule, mycolactone, interferes with T cell activation by inhibiting TCR signaling through an as yet unknown mechanism (41), underscoring T cell activation -and by inference IS assembly- as a relevant target for T cell disabling by *M. tubercolosis*.

Other pathogens have been reported to disrupt specific steps in TCR signaling. One such example is *Yersinia pestis*, which terminates TCR signaling using one of its outer membrane proteins, the protein tyrosine phosphatase YopH, that dephosphorylates key TCR signalosome components, including Lck, LAT and SLP-76 (64–67). *Bordetella pertussis* and *Bacillus anthracis* also suppress TCR signaling from its earliest step -activation of Lck- by elevating the cellular concentration of cAMP through their adenylate cyclase toxins, CyaA and edema toxin, respectively (70, 71). At variance, the *H. pylori* vacuolating cytotoxin (VacA) inhibits the Ca^2+^-calcineurin pathway that is responsible for the activation of the key transcription factor NF-AT by inducing plasma membrane depolarization through its anion channel activity (57, 58). Additionally, VacA perturbs TCR signaling through an independent pathway triggered by its receptor-binding moiety, which selectively enhances the activity of the MAP kinase p38 but not Erk, leading to a dysfunctional MAP kinase network (57). That these effects have the potential to target the IS is witnessed by the ability of *Bordetella pertussis* CyaA to impair IS assembly through local cAMP production (71, 72) (**Figure 2**, **Table 1**).

#### 3.2.3 Targeting the Actin Cytoskeleton

IS assembly is coordinated by the interplay of the actin and tubulin cytoskeletons. F-actin reorganization regulates multiple steps of IS formation, from integrin-mediated T cell adhesion to its cognate APC, to the recruitment of TCR microclusters to the cSMAC, to centrosome polarization beneath the synaptic membrane, to the process of sorting of cargoes, including TCRs, from early endosomes for their recycling to the IS to sustain signaling (94). Bacterial pathogens are masters at exploiting the host cell actin cytoskeleton for engulfment by host cells and intercellular dissemination, as exemplified by *Shigella flexneri*, *Yersinia pestis* and *Salmonella enterica* serovar *Typhimurium*. This is achieved by a remarkable array of T3SS effectors that promote actin remodeling by targeting directly or indirectly the Rho GTPases. The strategies evolved to modulate the activity of these small GTPases are multifarious, ranging from Rho GEF mimics (e.g. *Salmonella* SopB and SopE), to GAP mimics (e.g. *Salmonella* SptP, *Yersinia* YopE), to direct modulators of the active (GTP-bound) or inactive (GDP-bound) forms of Rho GTPases (e.g. the ADP-ribolysating *Clostridium* C3 toxin; the *Yersinia* protease YopT), to the process of F-actin filament assembly (e.g. *Shigella* IcsA and *Listeria* ActA mimicking activators of the actin nucleator N-WASP and of the actin adaptor Arp2/3, respectively; *M. tubercolosis* MtSerB2-mediated dephosphorylation and activation of cofilin) (95, 96). By acting on F-actin remodeling, these bacterial pathogens have the potential to interfere with the highly regulated process of IS assembly.

Direct experimental evidence in support of this hypothesis has been recently generated. *Shigella* had been previously shown to directly impair T cell chemotaxis through its T3SS effector IpgD, a lipid phosphatase that hydrolyses PI(4,5)P2, thus preventing leading edge formation in which actin dynamics plays a pivotal role (76). Recently Samassa and colleagues demonstrated that *Shigella* promotes actin polymerization in CD4^+^ T cell through an as yet unidentified T3SS effector which leads to an increase in cell stiffness, thereby impairing the ability of T cells to scan APCs for the presence of specific pMHC and hence affecting the efficiency of T cell:APC conjugate formation, which is sets the stage for IS assembly (77). Since other bacterial pathogens may exploit their T3SS system to invade, albeit not productively infect, T cells, they might exploit the actin-subverting effectors to similarly affect IS formation. A similar scenario can be hypothesized for the *H. pylori* vacuolating cytotoxin VacA, which binds T cells by interacting with the integrin LFA-1 (59) and triggers the activation of the Rho family guanine nucleotide exchanger Vav1 and the downstream activation of Rac1, leading to perturbations in the actin cytoskeleton (57).

F-actin reorganization during IS assembly is critically controlled by the dynamic redistribution of lipid kinases and phosphatases that generate local pools of specific phosphoinositides. Actin clearance from the IS center is required to generate the secretory domain where exocytic and endocytic events occur. This is regulated by depletion from the IS center of the lipid kinase PIP5K, which is required to replenish PI(4,5)P2 at the synaptic membrane, thus sustaining actin polymerization (97). Remarkably, modulation of phosphoinositide signaling is a major target shared by a variety of bacterial pathogens (98). An interesting example is the *M. tubercolosis* lipid phosphatase SapM, which dephosphorylates PI(4,5)P2 and PI3P to regulate the early stages of microbial phagocytosis and phagosome formation (35). Of note, while PI(4,5)P2 is implicated in F-actin polymerization during IS assembly, PI3P plays a crucial role in endosome trafficking, which is also centrally implicated in IS assembly, as detailed in the following section (**Figure 2**, **Table 1**).

#### 3.2.4 Targeting Vesicular Trafficking

T cell activation requires TCR signaling to be sustained for several hours (99). This is achieved through the sequential mobilization of two TCR pools associated with the plasma membrane and recycling endosomes, respectively (17–19). Translocation of the centrosome towards the T cell:APC contact sets the stage for the polarized delivery of endosomal TCRs through their dynein-dependent transport along the microtubules. This strategy is co-exploited by a number of other receptors as well as membrane-associated signaling mediators that modulate the TCR signaling cascade (11, 12).

Vesicular trafficking is widely highjacked by bacterial pathogens for infection as well as to disable the bactericidal mechanisms of phagocytes. Major targets in this process are the Rab GTPases, largely through the modulation of their activity by a variety of virulence factors that act as GAPs or GEFs on specific Rab family members (100). Examples of bacterial Rab GAPs are *M. tubercolosis* Ndk (43), *Salmonella enterica* SopD2 (53), *Legionella pneumophila* LepB (80) and *Shigella* VirA (78), while examples of bacterial Rab GEFs are *Legionella pneumophila* Lgp0393 (82) and DrrA/SidM (81). Additionally, as mentioned in the previous paragraph, phosphoinositide signaling, which is essential for endosome maturation through recruitment of Rab proteins or their regulators or effectors, is disrupted by phosphoinositide-specific virulence factors, such as the phosphoinositide phosphatases *M. tubercolosis* SapM and *Salmonella* enterica SopB (98). Hence, similar to phagocytes, these factors may be expected to interfere with vesicular trafficking in T cells, thereby impacting on IS assembly and function.

Strong support to this hypothesis has been provided by the finding that *Shigella* impairs IS assembly by disrupting the polarized recycling of TCR-containing endosomes to the IS through two T3SS effectors, the Rab1 GAP VirA and the Arf/Arl targeting cysteine protease IpaJ (77). Additionally, we have shown that forced expression of the *Salmonella* protease GtgE, which cleaves and inactivates Rab29 and Rab8 (101, 102), similarly impairs IS assembly by inhibiting two sequential steps in the vesicular transport pathway that regulates polarized TCR recycling to the IS (54). Of note, the activity of Rab32 is also modulated by *Salmonella* SopD2 acting as a GAP (53), highlighting a combined targeting of Rab29 by distinct virulence factors of this pathogen. A different strategy is exploited by *Bordetella pertussis*, which uses its adenylate cyclase toxin CyaA to impair recycling of the integrin LFA-1, leading to premature IS disassembly (71) (**Figure 2**, **Table 1**).

## 4 Conclusions and Outlook

Pathogens are masters in the art of spotting the vulnerabilities of target cells and evolve strategies to either neutralize or subvert these to their own advantage to infect target cells and evade immune mediated destruction. As the platform where the T cell response to antigen recognition is coordinated, the IS represents one of such vulnerabilities. This is witnessed by evidence accumulated over the past several years showing that the processes that regulate IS assembly, from TCR signaling, to cytoskeleton dynamics, to vesicular trafficking, are targeted by lymphotropic viruses to thwart the antiviral T cell response and infect neighboring cells while remaining undetectable (86, 87). Interesting, IS targeting is exploited also by tumor cells to suppress antitumor immunity through both contact-dependent and -independent mechanisms, as amply documented in chronic lymphocytic leukemia (103). Hence, it is not surprising that bacterial pathogens have co-opted this strategy to evade T cell mediated immunity. While the evidence supporting this notion is as yet scant, it is likely to represent only the tip of the iceberg since the cellular processes known to be disrupted or subverted by bacterial virulence factors that coordinate infection of target cells, such as cytoskeletal dynamics, membrane trafficking or phosphoinositide signaling, are also centrally implicated in the process of IS assembly. Hence studies focusing on the IS as target of bacterial virulence factors are expected to provide major insights into the mechanisms of immune evasion by bacterial pathogens. Of note, bacterial pathogens that infect cells that are transported to peripheral lymphoid tissues, such as DCs or macrophages, can interfere with priming pathogen-specific T cells. While pathogens that remain confined in infected tissues may influence T cell priming through soluble factors that can be transported by the lymph, their physical separation prevents them from directly deploying the full array of virulence factors, targeting rather APCs for targeting this process. However, naive T cells differentiated to helper or cytotoxic effectors are recruited to the site of infection to coordinate the fight against the pathogens in concert with the innate immune cells. Since effector T cells assemble immune synapses with target cells for the selective delivery of cytokines and cytotoxic molecules, the potential IS-modulating functions of bacterial virulence factors may be highly effective to evade the effector mechanisms of these cells.

## Author Contributions

NC and CB wrote the paper. NC prepared the artwork.

## Funding

CB is supported by grants from EU (ERC Synergy Grant 951329), AIRC (IG-2017 - ID 20148) and 464 Ministero dell’Istruzione, dell’Università e della Ricerca (Grant PRIN bando 2017 - 2017FS5SHL).

## Conflict of Interest

The authors declare that the research was conducted in the absence of any commercial or financial relationships that could be construed as a potential conflict of interest.

## Publisher’s Note

All claims expressed in this article are solely those of the authors and do not necessarily represent those of their affiliated organizations, or those of the publisher, the editors and the reviewers. Any product that may be evaluated in this article, or claim that may be made by its manufacturer, is not guaranteed or endorsed by the publisher.
